# Maternal Dexamethasone Exposure Alters Synaptic Inputs to Gonadotropin-Releasing Hormone Neurons in the Early Postnatal Rat

**DOI:** 10.3389/fendo.2016.00117

**Published:** 2016-08-31

**Authors:** Wei Ling Lim, Marshita Mohd Idris, Felix Suresh Kevin, Tomoko Soga, Ishwar S. Parhar

**Affiliations:** ^1^Brain Research Institute, School of Medicine and Health Sciences, Monash University Malaysia, Petaling Jaya, Malaysia

**Keywords:** glucocorticoid, GnRH neuron, preoptic area, synapsin I, reproduction

## Abstract

Maternal dexamethasone [(DEX); a glucocorticoid receptor agonist] exposure delays pubertal onset and alters reproductive behavior in the adult offspring. However, little is known whether maternal DEX exposure affects the offspring’s reproductive function by disrupting the gonadotropin-releasing hormone (GnRH) neuronal function in the brain. Therefore, this study determined the exposure of maternal DEX on the GnRH neuronal spine development and synaptic cluster inputs to GnRH neurons using transgenic rats expressing enhanced green fluorescent protein (EGFP) under the control of GnRH promoter. Pregnant females were administered with DEX (0.1 mg/kg) or vehicle (VEH, water) daily during gestation day 13–20. Confocal imaging was used to examine the spine density of EGFP–GnRH neurons by three-dimensional rendering and synaptic cluster inputs to EGFP–GnRH neurons by synapsin I immunohistochemistry on postnatal day 0 (P0) males. The spine morphology and number on GnRH neurons did not change between the P0 males following maternal DEX and VEH treatment. The number of synaptic clusters within the organum vasculosum of the lamina terminalis (OVLT) was decreased by maternal DEX exposure in P0 males. Furthermore, the number and levels of synaptic cluster inputs in close apposition with GnRH neurons was decreased following maternal DEX exposure in the OVLT region of P0 males. In addition, the postsynaptic marker molecule, postsynaptic density 95, was observed in GnRH neurons following both DEX and VEH treatment. These results suggest that maternal DEX exposure alters neural afferent inputs to GnRH neurons during early postnatal stage, which could lead to reproductive dysfunction during adulthood.

## Introduction

Gonadotropin-releasing hormone (GnRH) neurons regulate fertility in the vertebrates by stimulating the secretion of pituitary gonadotropins, thereby promoting gonadal hormone secretion and function. The unique developmental origin of GnRH neurons in the nasal region and the characteristic migration of GnRH neurons to the forebrain are well conserved across vertebrates (1–3). Within the rat hypothalamus, GnRH neurons are distributed along the medial septum (MS), diagonal band of Broca, preoptic area (POA), organum vasculosum of the lamina terminalis (OVLT), and anterior hypothalamic area (AHA), with fibers projecting to the OVLT and median eminence (4, 5). Approximately 1000–1300 GnRH neurons were found in the postnatal and adult rat brain (6, 7). However, abnormal migration and development of GnRH neurons in the brain during early stages is linked with hypogonadism and reproductive dysfunction or Kallmann syndrome in human (8, 9).

Morphological plasticity of GnRH neurons and synaptic inputs from various neuropeptides and neurotransmitters to GnRH neurons are modulated by the developmental stage, gender, hormonal, and environmental cues (10, 11). Immunohistochemical studies have identified morphologically smooth and spiny subtypes of GnRH neurons in the brain, and number of spiny GnRH neurons increase with pubertal maturation in male and female rats (12). Biocytin-filled GnRH neurons expressing GFP in mice show increased spine density on GnRH soma and dendrite in adult stages, indicating increased afferent inputs to GnRH neurons during pubertal maturation (13). Increase in somatic and dendritic spine density in subpopulation of GFP–GnRH neurons expressing cFos protein during preovulatory luteinizing hormone (LH) surge further implicates the role of gonadal steroids in modulating spine plasticity of GnRH neurons (14). In addition, changes in γ-amino butyric acid (GABA)-ergic and glutamatergic synaptic terminal appositions contacting GnRH neurons in rats are demonstrated during estradiol-induced LH surge and reproductive aging (15, 16).

Based on ultrastructural characterization, GnRH neurons receive few synaptic inputs on the soma and dendrites (17, 18); however, the presence of numerous spines along the extension of dendritic profiles in biocytin-filled GFP–GnRH neurons suggests that GnRH neurons receive substantially more neural inputs than previously known (19). Electrophysiological recordings of GnRH neurons in young and adult rats demonstrated that GnRH neuronal soma and dendrites are electrically active to initiate action potentials in the presence of voltage-gated channels (20, 21). Furthermore, GnRH neurons exhibit persistent tonic inhibition current, mediated by the activation of extrasynaptic GABA_A_ receptor δ subunit, important for the regulation of GnRH neuronal excitability (22). Taken together, integration of multiple internal and external cues is important for the proper synaptic network of GnRH neurons to modulate GnRH function.

Maternal exposure to stress and elevated glucocorticoid levels has deleterious effects on the brain development, neuronal function, and behavior of the offspring (23, 24). Gestational stress alters the dendritic length and branching of neurons in the sexually dimorphic medial POA of adult males, suggesting possible modification of the neuronal activity and sexual behavior during adulthood (25). Exposure to the glucocorticoid receptor agonist, dexamethasone (DEX), during fetal stages reduce plasma testosterone levels, alter sexual maturation and reproductive behavior in male rat offpring (26–28), as well as delay pubertal onset with irregular estrous cycle in female rodent offspring (29, 30). Taken together, these changes are attributed to the effect of glucocorticoid exposure during fetal stages along the hypothalamic–pituitary–gonadal axis during development.

In rodents, a subpopulation of GnRH neurons co-expresses the glucocorticoid receptor (31, 32), implicating that glucocorticoids have a direct effect on GnRH neurons and function within the hypothalamus. DEX exposure during early life reduces hypothalamic GnRH levels in male rats (26) and GnRH expression in female mice (30). Decreased GnRH gene expression and migratory capacity of GnRH cells by DEX occurs *in vitro* (32–34). Furthermore, maternal DEX exposure decreases the number of GnRH neurons and their branched dendritic morphology within the OVLT of early postnatal transgenic male rats expressing the enhanced green fluorescent protein (EGFP) under the control of GnRH promoter (35). This confirms the effect of DEX exposure on GnRH neuronal development in early-life stages.

We hypothesize that maternal DEX exposure may have an effect on the plasticity of GnRH neurons and the afferents interacting with GnRH neurons during early developmental stage, which may lead to reproductive dysfunction and sexual behavioral changes in adulthood. Therefore, this study aims to determine the effect of maternally administered DEX during late gestational stages on the spine development and synaptic input of early postnatal EGFP–GnRH neurons in the male offspring using confocal imaging on spine density along with a presynaptic marker, synapsin I (syn I), and a postsynaptic marker, postsynaptic density 95 (PSD-95), immunohistochemistry.

## Materials and Methods

### Animals

The transgenic Wistar rats expressing EGFP under the control of 3.0-kb rat GnRH promoter were used throughout this study (a gift from Masakatsu Kato and Yasuo Sakuma from University of Tokyo Health Sciences, Japan). The GnRH expression in the EGFP cells of these transgenic rats has been characterized (20, 36, 37). All rats were housed under constant temperature of 22°C and maintained on 12-h light:12-h dark cycle with access to food and water *ad libitum*. All procedures were conducted according to the Guidelines of the Animal Ethics Committee of Monash University (ethics approval number MARP/2011/064).

### Maternal DEX Administration

Adult female EGFP–GnRH transgenic rats were time-bred and designated as embryonic day 0 (E0) with presence of spermatozoa in the vaginal smear. DEX-21-phosphate disodium salt (DEX; Sigma, USA) was suspended in sterile distilled water and administered *via* subcutaneous injection at a dose of 0.1 mg/kg body weight (27) daily from gestation day 13–20. The control group (VEH) received an equivalent volume of distilled water. The day of birth was designated as postnatal day 0 (P0), and litters of eight or more pups were included in this study. P0 male offspring of maternally VEH- (VEH-P0, *n* = 6) and DEX-administered (DEX-P0, *n* = 6) group were taken from two different litters for each group. Sex determination of the P0 pups was confirmed by expression of the male-specific sex-determining region-*Y* (*Sry*) gene as previously described (35).

### Tissue Preparation

The P0 male pups of maternally VEH- and DEX-administered were anesthetized with Zoletil–Ketamine–Xylazine (13.5 mg/kg) and transcardially perfused with 0.1M phosphate buffer (PB) saline (PBS, pH 7.4) containing 1% heparin, followed by cold 4% paraformaldehyde (PFA) dissolved in 0.1M PB. The brains were processed as described (35) and rapidly frozen in powdered dry ice to be stored at −80°C. The brain tissues were sectioned sequentially into four series in the coronal plane at 60 μm thickness from the MS to the anterior hypothalamus region (Bregma +1.56 to −1.44 mm) (38) using a cryostat. The sections were further processed for spine density analysis, syn I, and PSD-95 immunohistochemistry.

### Spine Density Imaging

Two alternate series of brain sections from each VEH-P0 and DEX-P0 males were processed for spine density analysis of the EGFP–GnRH neurons. The brain sections were washed in 0.1M PBS and pasted onto 3-aminopropylsilane-coated slides (SuperFrost Plus, Fisher Scientific, USA), and coverslips were applied with mounting medium (Vectashield, Vector Laboratories, USA).

The EGFP–GnRH transgenic rats expressing high levels of EGFP allows visualization of fine details of the GnRH neuronal morphology that are not apparent after GnRH and GFP immunohistochemistry (37). GFP immunostaining of EGFP–GnRH neurons of P0 stages show weak staining and localization to the nucleus compared to GFP staining in the soma and dendrites of adult stages (35). Therefore, confocal imaging of the EGFP–GnRH neurons allows direct visualization of protrusions emerging from the GnRH neuronal soma and dendrites in P0 males.

The total number of EGFP–GnRH neurons acquired from each VEH-P0 and DEX-P0 male pups was approximately 25–30 cells from 1 to 2 sections of the MS, OVLT, and AHA (*n* = 6/group) regions, respectively. All the visible EGFP–GnRH neurons with soma and dendrites present within the tissue section were analyzed. Individual GnRH neurons were scanned using the laser scanning confocal microscope (C1si, Nikon, Japan) equipped with NIS elements AR software version 4.0 (Nikon, Japan), at 488 nm excitation wavelength for detection of EGFP. Each GnRH neuron was scanned using the 60× Plan Apo water immersion objective lens (numerical aperture 1.2) and 4× digital zoom. The EGFP–GnRH neurons for spine density analysis were captured at a higher laser power compared to that of syn I-ir images to detect the spines and filopodia on the cell body and dendrites. The frame size for each GnRH neuron captured was set to 1024 × 1024 pixels and comprised of 80–150 stacks of serial images taken at 0.2 μm intervals through the entire depth of each neuron. The cell body containing the initial portion of the proximal dendrites were captured for each EGFP–GnRH neuron.

### Spine Density Analysis

For the analysis of spine density, the images of each EGFP–GnRH neuron were subjected to binary threshold settings using the Nikon NIS elements AR software, such that the spines and filopodia emerging from the cell body and proximal dendrites were included in the binary images. The same threshold values were applied to all images of EGFP–GnRH neurons analyzed. The binary images were converted into three-dimensional (3-D) images and further processed into video images with rotation at the *Y*-axis. Using the 3-D video, number of spines and filopodia were counted on the soma and along the initial 20–30 μm length of primary dendrites for each GnRH neuron. Spines were identified as protrusions of length <5 μm extending from the soma or dendrites, while protrusions of >5 μm were classified as filopodia (13).

Spine density along the secondary dendrite of GnRH neurons with bipolar or complex morphology was not analyzed. GnRH neurons with bipolar morphology display shorter (<50 μm) secondary dendrites, and their active regions with higher number of spines and synapses are located proximally along the primary dendrites (13). The number of spines and filopodia emerging from each GnRH soma and dendrite was combined to provide the mean value of spine and filopodia number per GnRH neuron for each VEH-P0 and DEX-P0 males. The grand mean values for the spine and filopodia number per GnRH neuron were compared between the VEH-P0 and DEX-P0 group, respectively. The investigator was blinded to the treatment group for binary processing and analysis of spine and filopodia on the GnRH neurons.

### Synapsin I and PSD-95 Immunofluorescence

Two series of brain sections from each VEH-P0 and DEX-P0 male were processed for syn I and PSD-95 free-floating immunocytochemistry. The brain sections covering the entire POA area (*N* = 13–14 sections/sample) were washed in 0.1M PBS, incubated in blocking solution (0.1% Triton-X and 2.0% NGS in PBS) for 1 h, followed by incubation in rabbit polyclonal synapsin I antibody (1:500; AB1543P, Chemicon, USA) for 48 h at 4°C or rabbit polyclonal PSD-95 antibody (1:1000; AB18258, Abcam, UK) for 24 h at 4°C. The sections were then incubated with biotinylated goat anti-rabbit IgG (1:200; Vectastain ABC Elite kit, Vector Laboratories, USA), followed by avidin-biotinylated horseradish peroxidase complex (20 μl/ml) and streptavidin coupled to AlexaFluor 594 (1:300, Molecular Probes, USA) and processed as previously described (35) for analysis.

### Antibody Characterization

The rabbit polyclonal anti-synapsin I antiserum was raised against a mixture of synapsin Ia and Ib purified from bovine brain, which recognizes two bands of 77 and 80 kDa on Western blot analyses. Immunolabelling is also blocked by preadsorption of the antibody with synapsin I according to the manufacturer’s technical information. The syn I immunoreactive (syn I-ir) punctae staining in this study was similar to the staining using other syn I antibodies (39, 40). Controls for the syn I and PSD-95 staining involved the omission of primary antibody in the initial step, which resulted in the absence of punctae staining on brain sections from each group.

### Synapsin I and PSD-95 Confocal Imaging

The EGFP-expressing GnRH neurons located within the MS, OVLT, and AHA were captured to determine the number of close apposition of syn I-ir punctae cluster on the GnRH neurons for each group. The total number of EGFP–GnRH neurons acquired from each VEH-P0 and DEX-P0 male pups were approximately 25–30 cells from 1 to 2 sections of the MS, OVLT, and AHA (*n* = 6/each group) regions, respectively. Every visible EGFP–GnRH neuron with soma and dendrites present within tissue sections of the MS, OVLT, and AHA was analyzed for the syn I-ir cluster apposition on the GnRH neurons. Therefore, discrepancy in the total number of GnRH neurons analyzed for the VEH-P0 and DEX-P0 males in the OVLT and AHA was not due to random sampling or bias. Concurrently, the EGFP–GnRH neurons within the OVLT area were observed for the localization of PSD-95-ir punctae cluster. Approximately five visible EGFP–GnRH neurons with presence of soma and dendrites were analyzed for the PSD-95-ir punctae staining from 1 to 2 sections of the OVLT and AHA region of VEX-P0 and DEX-P0 males (*n* = 3/each group).

The individual GnRH neurons were scanned using laser scanning confocal microscope (C1si, Nikon) at 488 and 543 nm excitation wavelengths for detection of EGFP–GnRH and syn I-ir or PSD-95-ir puncta, respectively. Each GnRH neuron was scanned using the 60× Plan Apo water immersion objective lens (numerical aperture 1.2) with 4× digital zoom. Scans at each wavelength were performed sequentially across the optical sectioning to avoid bleed-through between the channels. The frame size of each EGFP–GnRH neurons captured was set to 1024 × 1024 pixels, comprised of 50–80 stacks of serial images taken at 0.2 μm intervals through the entire depth of each neuron. Images containing the cell body with initial portion of the proximal dendrites were captured for each EGFP–GnRH neuron from each group.

### Synapsin I and PSD-95 Punctae Cluster Analysis

The syn I-ir and PSD-95-ir punctae clusters exhibiting close apposition with EGFP–GnRH cell body or primary dendrite were manually determined by scanning through the individual *z*-slices using the NIS elements AR software. syn I-ir and PSD-95-ir punctae clusters were defined as being in contact with the EGFP–GnRH neurons if there were no pixel visible between the green and red fluorescence (39, 41). The length of the primary dendrite of EGFP–GnRH neurons captured within the frame size was less than 40 μm from the soma, depending on the orientation of neurons, morphological subtypes, and EGFP expression along the dendrites. Therefore, the number of syn I-ir and PSD-95-ir appositions was counted on the soma and along initial 20–30 μm length of primary dendrite, as the primary dendrite was defined as dendritic projections having the greatest diameter exiting the cell body (13). For GnRH neurons exhibiting bipolar or complex morphology, the syn I-ir and PSD-95-ir contacts along the secondary dendrite were not included. Close appositions of the syn I-ir and PSD-95-ir punctae cluster on GnRH soma and primary dendrite were confirmed by 3-D rotation of the cells using the NIS elements AR software. The investigator was unaware of the particular treatment when analyzing the GnRH neurons. The number of syn I-ir and PSD-95-ir contacts on the soma and primary dendrites of each GnRH neuron was combined to provide the mean values to form the total number of syn I-ir and PSD-95-ir appositions per animal for each group. The grand mean values for syn I-ir and PSD-95-ir contacts on GnRH neuronal soma and primary dendrites were compared between the VEH-P0 and DEX-P0 group, respectively.

The size of syn I-ir punctae cluster in close apposition with the GnRH soma and dendrites was further measured to determine whether levels of syn I cluster contacting the GnRH neurons in the OVLT and AHA region differ between VEH-P0 and DEX-P0 males. Images of each syn I-ir punctae cluster showing close apposition with the GnRH soma and primary dendrites from the 3-D orthogonal view of single optical section (0.2 μm) on the NIS Elements AR software were captured and saved as tiff file (RGB mode). The size of each syn I-ir synaptic cluster along the *X–Y, Y–Z*, and *X–Y* axes of the single optical section were measured using Image Pro-Plus version 6.0 (Media Cybernetics Incorporation, USA). The red channel to detect the syn I-ir signal was selected with a constant range of intensity threshold value to determine the number of pixels corresponding to the syn I-ir cluster area in close contact with GnRH soma and dendrite. These values were combined to provide mean number of syn I-ir pixel per cluster for each GnRH neuron within individual animals in a treatment group, and the grand mean values were compared between the VEH-P0 and DEX-P0 group.

For each GnRH neuron analyzed, the *z*-series of confocal images were converted into projected stacks and saved as tiff files (RGB mode). The images of each GnRH neuron were used to quantify the number of syn I-ir clusters in areas adjacent to the GnRH neurons, but not directly opposed to them, using the Image Pro-Plus. The red channel representing the syn I-ir punctae cluster was selected with constant range of intensity threshold value and size of syn I-ir cluster for each image analyzed. The total number of syn I-ir punctae clusters in each image was determined by taking the average number of syn I-ir clusters counted from two 400 μm^2^ square boxes in apposite areas adjacent to the GnRH neurons and expressed as number of syn I-ir cluster/400 μm^2^. The values were combined to provide the mean number of syn I-ir cluster for each animal, and the grand mean values were compared between the VEH-P0 and DEX-P0 group.

### Statistical Analysis

All data are represented as mean ± SEM, and statistical analysis were carried out using the PASW Statistic 18 (SPSS Inc., Chicago, IL, USA). Statistical differences in the number of spine and filopodia emerging from EGFP–GnRH neurons and PSD-95-ir on GnRH neurons between VEH-P0 and DEX-P0 males were tested using unpaired Student’s *t*-test for each region. Statistical significance was determined using two-way analysis of variance (ANOVA) for the total number of syn I-ir contacts per GnRH neurons and syn I-ir cluster in areas adjacent to the GnRH neurons using region and treatment as factors. The significant main effects or interactions from two-way ANOVA were further analyzed using unpaired Student’s *t*-tests. The number of syn I-ir punctae cluster exhibiting close apposition with each GnRH soma and dendrite and the number of syn I-ir pixel per cluster in contact with each GnRH neuron of the VEH-P0 and DEX-P0 group were tested using unpaired Student’s *t*-test. *P* < 0.05 was considered as significant difference.

## Results

### Analysis of Spine Density in EGFP–GnRH Neurons

**Figure 1:**
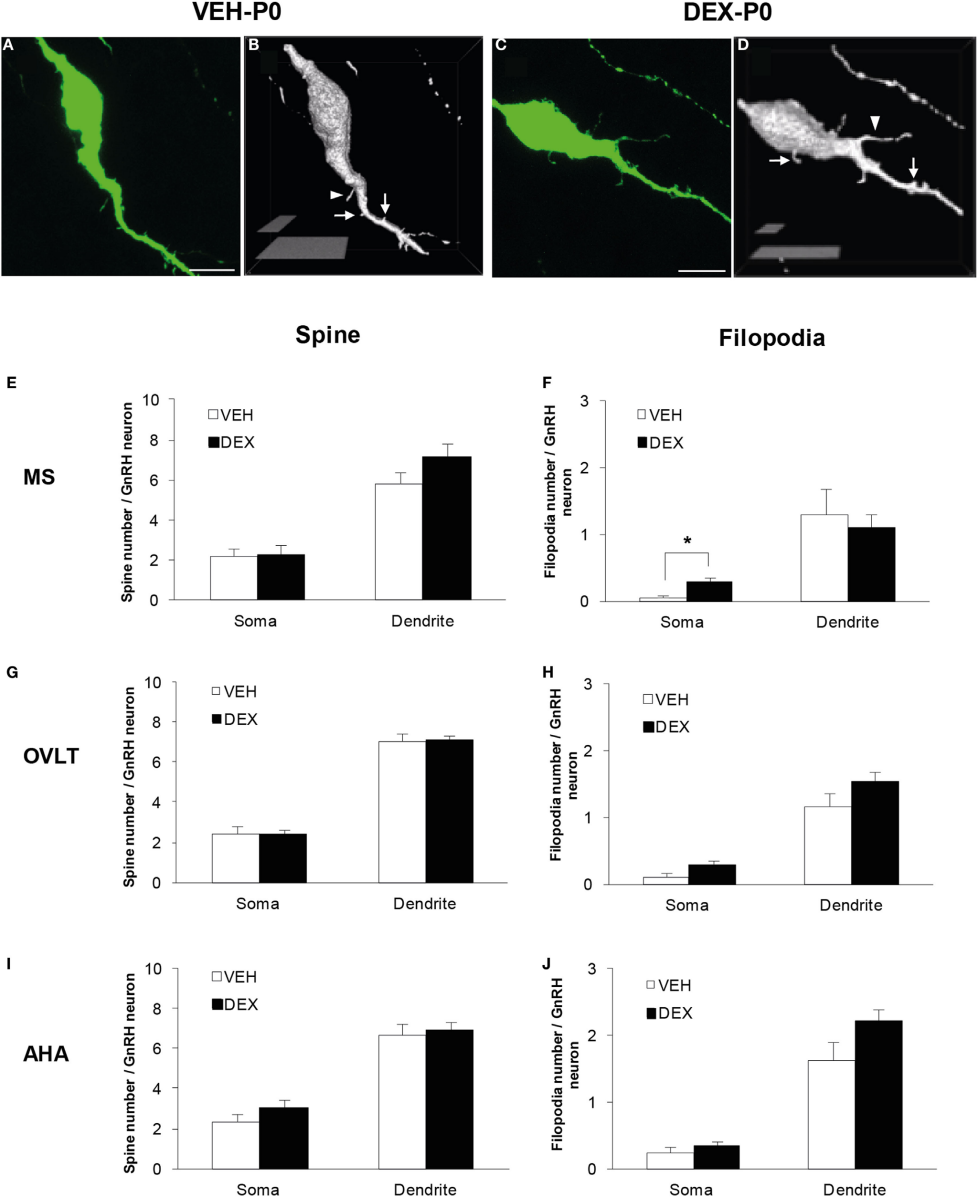
**Spine density analysis of enhanced green fluorescent protein (EGFP)-expressing gonadotropin-releasing hormone (GnRH) neurons from P0 males of maternally vehicle (VEH-P0)- and dexamethasone-treated (DEX-P0)**. Projected confocal images and binary processing for three-dimensional rendering of EGFP–GnRH neurons of **(A,B)** VEH-P0 and **(C,D)** DEX-P0 males showing spines (*white arrow*) and filopodia (*white arrowhead*) on the GnRH neuronal soma and along the primary dendrite. The number of spines emerging from the soma and primary dendrite of GnRH neurons in the MS **(E)**, OVLT **(G)**, and AHA **(I)** did not differ between VEH-P0 and DEX-P0 males (*n* = 6/group). Small increase in the number of filopodia on the GnRH neuronal soma was found in the MS **(F)** of DEX-P0 males. However, the number of filopodia emerging from the soma and primary dendrite of GnRH neurons in the OVLT **(H)** and AHA **(J)** region did not differ between VEH-P0 and DEX-P0 males. MS, medial septum; OVLT, organum vasculosum of the lamina terminalis; AHA, anterior hypothalamic area. Data are represented by the mean ± SEM for each group. ***P < 0.05 compared to VEH-P0 group. Scale bar = 10 μm **(A,C)**, 5 μm, and 20 μm **(B,D)**.

Confocal imaging of the EGFP–GnRH neurons without amplification of GFP signal enabled direct visualization of protrusions emerging from the GnRH soma and dendrites in P0 males (Figures 1A,C). Binary processing and 3-D video rotation of the confocal images were used for quantitative analysis of spines on the VEH-P0 and DEX-P0 GnRH neurons (Figures 1B,D). The EGFP–GnRH neurons from VEH-P0 and DEX-P0 males were observed to exhibit spines and filopodia extending from the soma and along the primary dendrites.

Quantitative analysis of spine and filopodia numbers were performed on GnRH neurons of VEH-P0 and DEX-P0 males (*n* = 6/group) located within the MS (VEH-P0, *n* = 39 cells; DEX-P0, *n* = 42 cells), OVLT (VEH-P0, *n* = 78 cells; DEX-P0, *n* = 83 cells), and AHA (VEH-P0, *n* = 34 cells; DEX-P0, *n* = 38 cells) region (Figures 1E–J). The number of spines, identified as protrusions less than 5 μm in length, emerging from the GnRH neuronal soma and along the primary dendrite, respectively, did not differ in the MS (Figure 1E), OVLT (Figure 1G), and the AHA (Figure 1I) region of VEH-P0 and DEX-P0 males. The number of filopodia, emerging from the GnRH neuronal soma within the MS region (Figure 1F), was higher in the DEX-P0 males (VEH-P0: 0.03 ± 0.03, DEX-P0: 0.30 ± 0.07; *P* < 0.05), but not along the primary dendrite. However, the number of filopodia emerging from the GnRH neuronal soma and primary dendrite in the OVLT (Figure 1H) and AHA (Figure 1J) did not differ between the VEH-P0 and DEX-P0 males.

### Synapsin I Immunofluorescence in EGFP–GnRH Neurons

**Figure 2:**
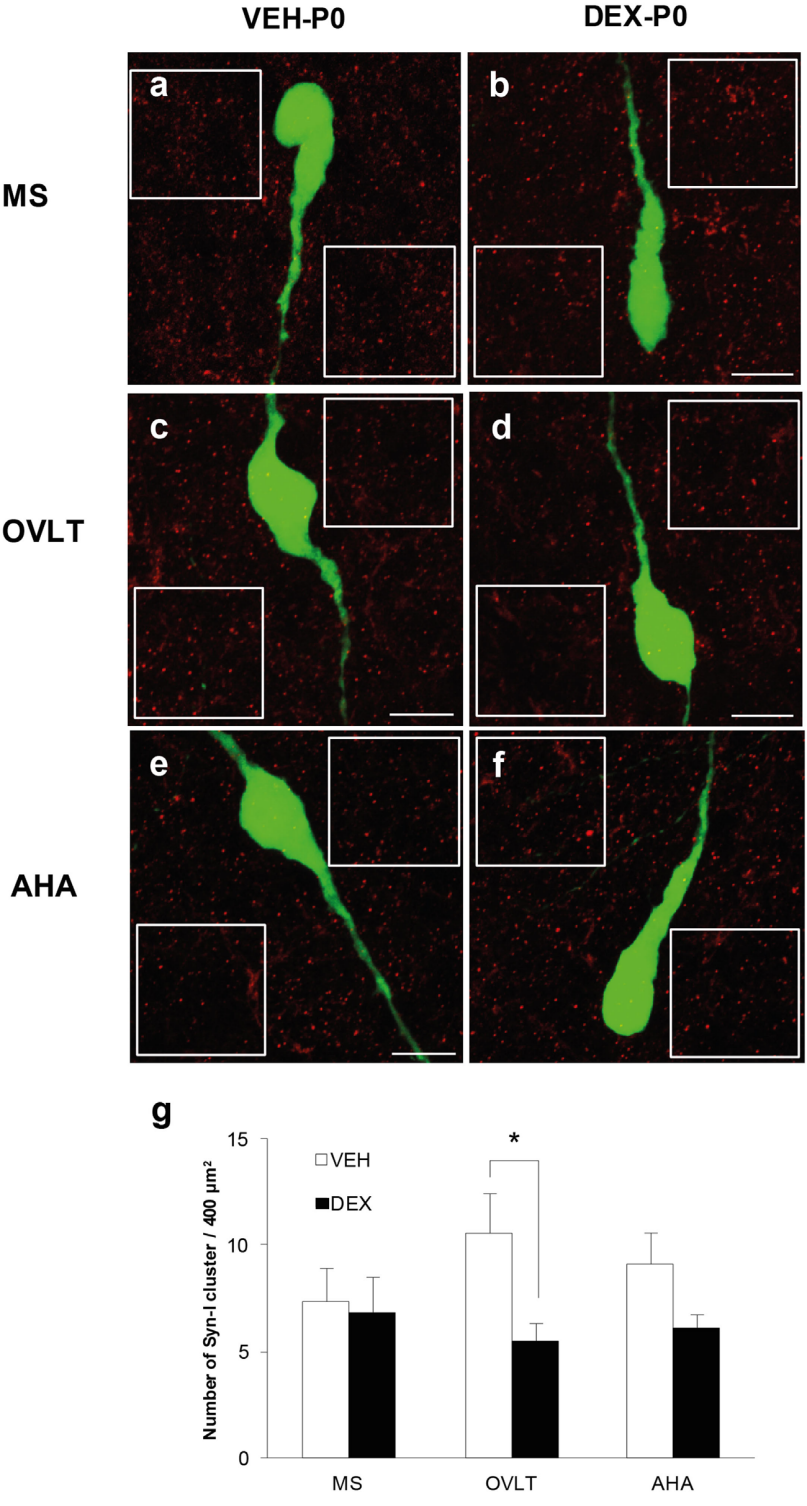
**Quantification of synapsin I-immunoreactive punctae cluster (syn I-ir, *red*) in areas adjacent to the EGFP–GnRH neurons (*green*)**. Representative photomicrographs for the quantification of average number of syn I-ir punctae from two 400 μm^2^ areas (*white box*) placed at both sides of each neuron, depending on each neuronal orientation in the image plane, for each VEH-P0 and DEX-P0 GnRH neurons analyzed in the MS **(A,B)**, OVLT **(C,D)**, and AHA **(E,F)**. The average number of syn I-ir punctae cluster **(G)** was decreased in the OVLT region of DEX-P0 compared to the VEH-P0 males. Data are represented by the mean ± SEM for each group. *P < 0.05 compared to VEH-P0 group. Scale bar = 10 μm **(A–F)**.

Immunofluorescence of syn I-ir inputs revealed punctae cluster in close apposition to the EGFP–GnRH neurons as well as in areas surrounding the EGFP–GnRH neurons within the MS (Figures 2A,B), OVLT (Figures 2C,D), and AHA (Figures 2E,F) of VEH-P0 and DEX-P0 males. The number of syn I-ir punctae was assessed in areas adjacent to the GnRH neurons to examine the synaptic clusters in the vicinity of GnRH neurons within the MS, OVLT, and AHA region of VEH-P0 and DEX-P0 males (Figure 2G). Two-way ANOVA analysis (treatment × region factor) showed that maternal DEX treatment had a significant effect [*F* (1, 30) = 5.84; *P* < 0.05] on the average number of synaptic cluster inputs surrounding the GnRH neurons. No significant region or interaction effects were found for the number of syn I-ir inputs surrounding the GnRH neurons. Maternal DEX exposure significantly decreased the average number of synaptic inputs surrounding the GnRH neurons within the OVLT region (VEH-P0: 10.6 ± 1.9, DEX-P0: 5.5 ± 0.9; *P* < 0.05). However, the number of synapses in areas adjacent to the GnRH neurons located within the MS and AHA region was not altered by maternal DEX exposure.

**Figure 3:**
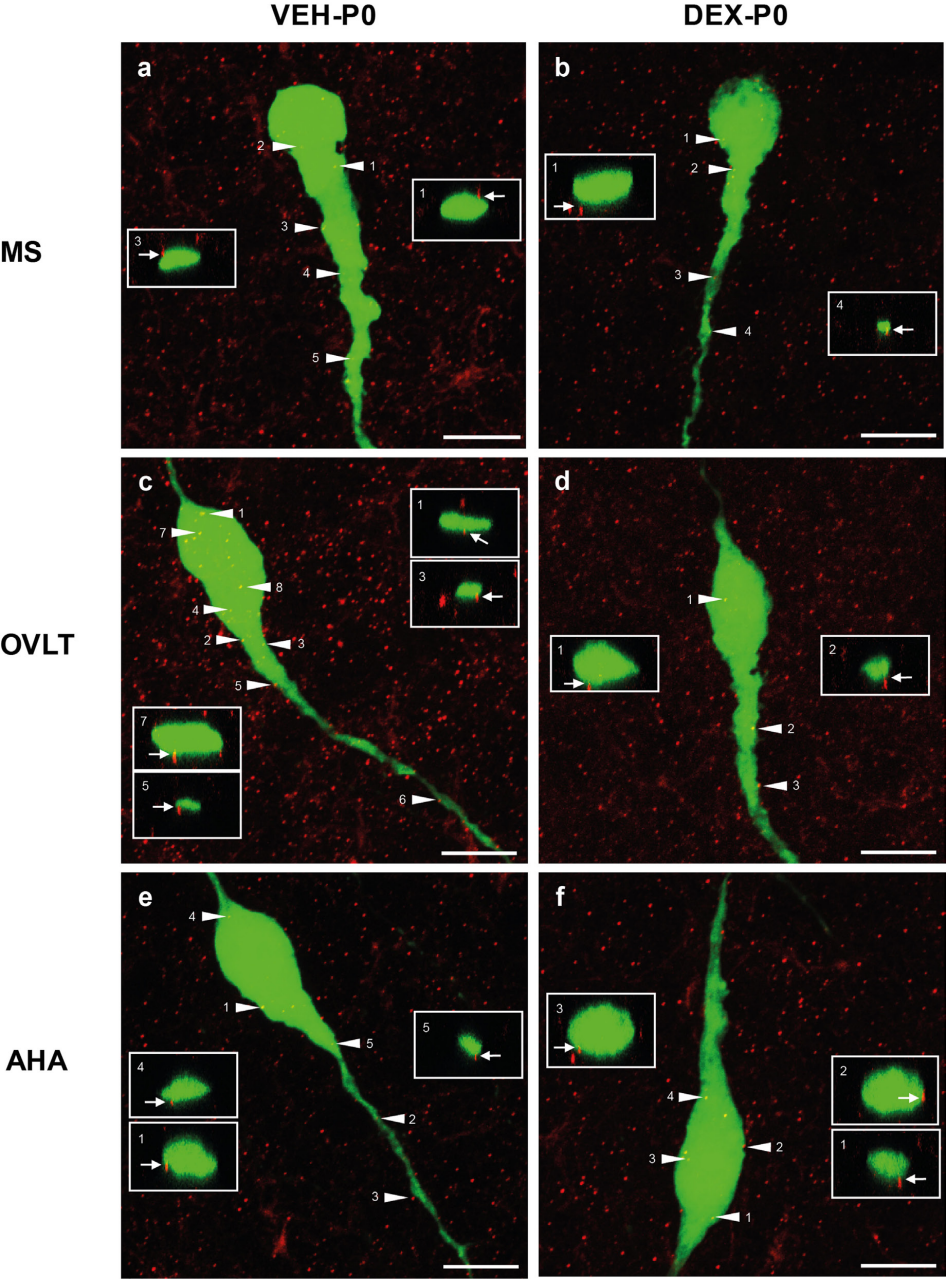
**Photomicrographs of syn I-ir punctae cluster (*red*) on the EGFP–GnRH (*green*) neuronal soma and primary dendrite of the VEH-P0 and DEX-P0 males in MS (A,B), OVLT (C,D), and AHA (E,F) region**. Boxed region (*white*) of syn I-ir cluster was taken along the *X–Z* axis from the orthogonal view of single optical sections (0.2 μm) with specific syn I-ir inputs identified by the numbers (*white arrowhead*) to illustrate the close appositions of the syn I-ir punctae cluster (*white arrow*) on the EGFP–GnRH neurons. Scale bar = 10 μm **(A–F)**.

Analysis of individual GnRH neurons using high magnification confocal images demonstrated numerous close appositions of syn I-ir punctae cluster on the EGFP–GnRH neuronal soma and along the primary dendrites in the MS (Figures 3A,B), OVLT (Figures 3C,D), and the AHA (Figures 3E,F) region of VEH-P0 and DEX-P0 males. Close apposition of syn I-ir inputs on the GnRH soma and primary dendrites was also demonstrated from the orthogonal projections (*X–Z* axis) of the corresponding single optical sections (0.20 μm) from the confocal stacks of the VEH-P0 and DEX-P0 EGFP–GnRH neurons.

**Figure 4:**
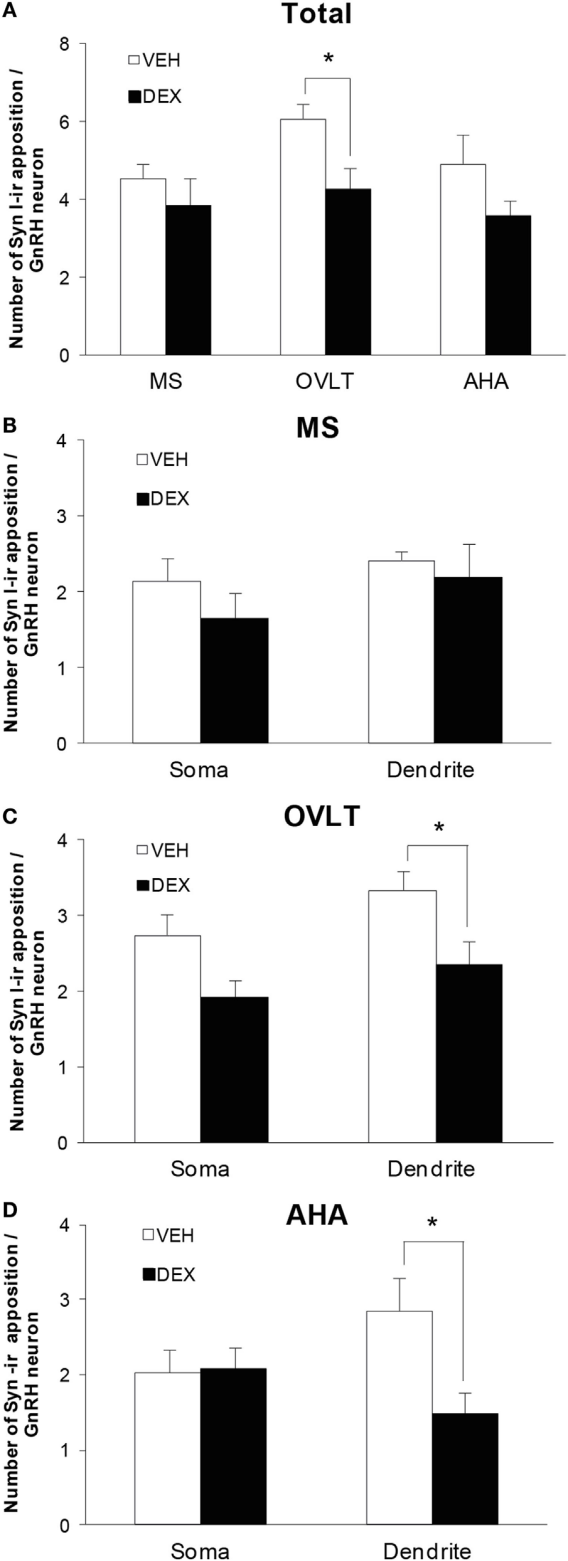
**Number of syn I-ir punctae cluster in close apposition with the EGFP–GnRH neurons of VEH-P0 and DEX-P0 males (*n* = 6/group)**. **(A)** Total number of syn I-ir punctae cluster on the EGFP–GnRH neurons was decreased in the OVLT region of DEX-P0 males. Numbers of syn I-ir punctae on soma and along primary dendrite of EGFP–GnRH neurons, respectively, were shown for GnRH neurons in the MS **(B)**, OVLT **(C)**, and AHA **(D)** region. Number of syn I-ir punctae cluster was decreased along the primary dendrite of EGFP–GnRH neurons in the OVLT and AHA region of DEX-P0 males. Data are represented by the mean ± SEM for each group. *P < 0.05 compared to VEH-P0 group.

Quantitative analysis of the total number of syn I-ir appositions on the GnRH neurons were combined from the number of syn I-ir punctae cluster on the GnRH neuronal soma and primary dendrite (Figure 4A) of the VEH-P0 and DEX-P0 males (*n* = 6/group) in the MS (VEH-P0, *n* = 44 cells; DEX-P0, *n* = 47 cells), OVLT (VEH-P0, *n* = 85 cells; DEX-P0, *n* = 72 cells), and AHA region (VEH-P0, *n* = 48 cells; DEX-P0, *n* = 35 cells). Two-way ANOVA analysis (treatment × region factor) revealed a significant effect of maternal DEX exposure [*F* (1, 30) = 8.34, *P* < 0.01] on the total number of syn I-ir appositions per GnRH neuron. No significant region or interaction effect was found on total number of syn I-ir inputs per GnRH neuron. Maternal DEX exposure significantly decreased the total number of synaptic inputs on GnRH neurons located within the OVLT (VEH-P0: 6.0 ± 0.4, DEX-P0: 4.3 ± 0.6; *P* < 0.05). In contrast, the synaptic inputs in close apposition with the GnRH neurons within the MS and AHA region were not affected by maternal DEX exposure.

The number of synaptic cluster inputs on GnRH neuronal soma and primary dendrite, respectively, was compared between the VEH-P0 and DEX-P0 group in the MS, OVLT, and AHA region (Figures 4B–D). Maternal DEX treatment decreased the number of syn I-ir cluster apposition along the GnRH primary dendrites (VEH-P0: 3.3 ± 0.2, DEX-P0: 2.3 ± 0.3; *P* < 0.05) within the OVLT region, but not the GnRH neuronal soma (VEH-P0: 2.7 ± 0.3, DEX-P0: 1.9 ± 0.2; *P* = 0.055). Within the AHA region, maternal DEX exposure decreased the number of syn I-ir cluster apposition along the primary dendrites (VEH-P0: 2.9 ± 0.4, DEX-P0: 1.5 ± 0.3; *P* < 0.05). However, the number of synaptic inputs on the soma and along the primary dendrites of GnRH neurons in the MS region was not altered by maternal DEX exposure.

**Figure 5:**
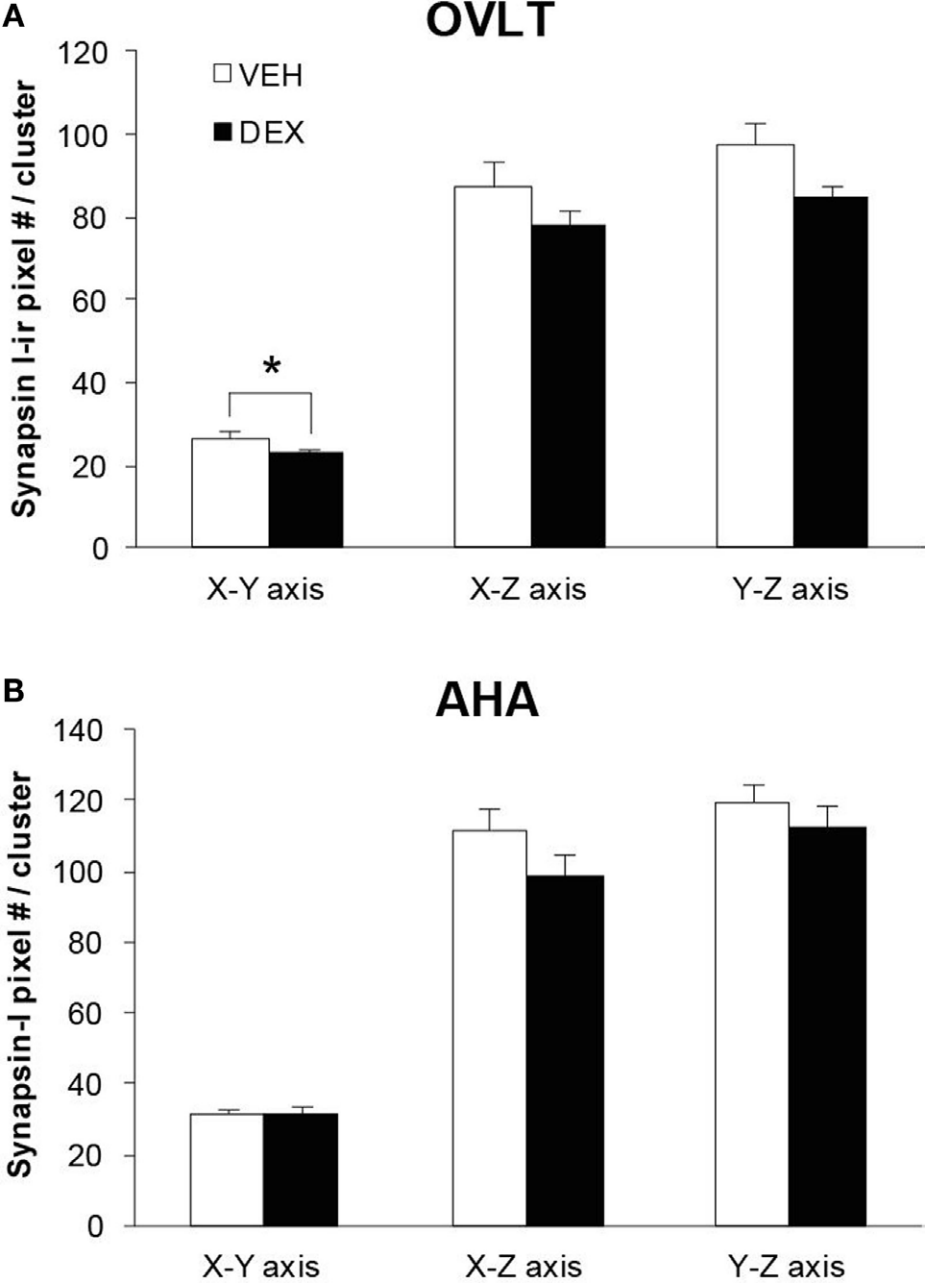
**Levels of syn I-ir punctae cluster in close apposition with the EGFP–GnRH neurons located in the OVLT (A) and AHA (B) region of VEH-P0 and DEX-P0 males**. The number of syn I-ir pixel per cluster of the EGFP–GnRH neurons, measured along the *X–Y* axis from single laser confocal optical sections, was lower in DEX-P0 males located within the OVLT region. *P < 0.05 compared to VEH-P0 group.

**Figure 6:**
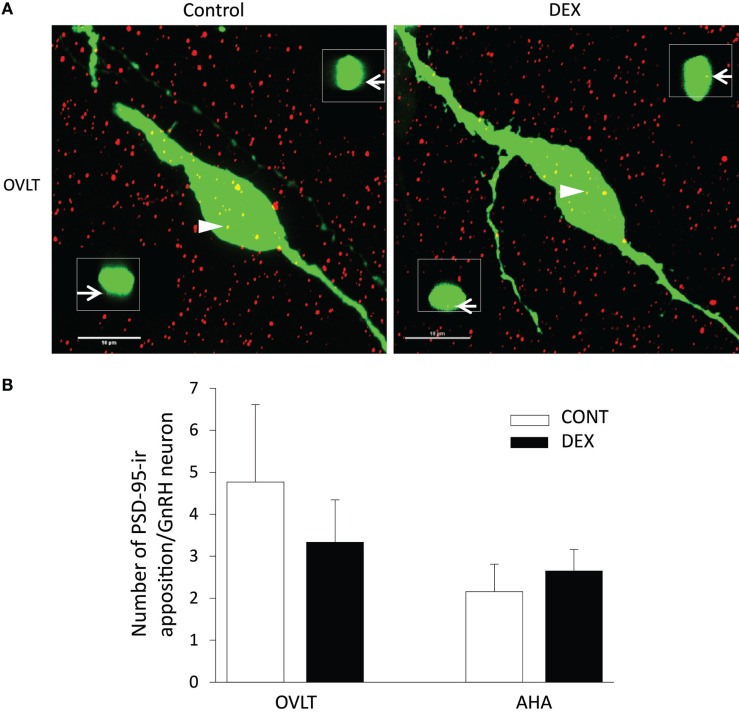
**Photomicrographs of PSD-95-ir (*red*) on the EGFP–GnRH (*green*) neuronal soma and primary dendrite of the VEH-P0 and DEX-P0 males in the OVLT (A)**. Boxed region (*white*) of PSD-95-ir was taken along the *X–Z* axis from the orthogonal view of single optical sections (0.2 μm) with specific PSD-95-ir inputs identified by the numbers (*white arrowhead*) to illustrate the close appositions of the PSD-95-ir (*white arrow*) on the EGFP–GnRH neurons. Numbers of PSD-95-ir inputs on soma and along primary dendrite of EGFP–GnRH neurons, respectively, were shown for GnRH neurons in the OVLT and AHA region **(B)**. Scale bar = 10 μm.

As the total number of syn I-ir punctae cluster in close apposition with the GnRH neurons was decreased within the OVLT region (Figure 4A), we determined whether the levels of syn I-ir cluster contacting the GnRH neurons was affected by maternal DEX exposure (Figures 5A,B). The number of syn I-ir pixel for each cluster in contact with the GnRH neuronal soma and primary dendrite was analyzed along the *X–Y, Y–Z*, and *X–Z* axes using single optical sections of the 3-D orthogonal view. The number of syn I-ir pixel per cluster of GnRH neurons measured at the *X–Y* axis was decreased in the OVLT region of DEX-P0 males (VEH-P0: 26.4 ± 1.2, DEX-P0: 22.8 ± 1.0; *P* < 0.05). However, the number of syn I-ir pixel per cluster of GnRH neurons at the *X–Z* axis and *Y–Z* axis did not differ between the VEH-P0 and DEX-P0 males. In the AHA region, the number of syn I-ir pixel per cluster of GnRH neurons measured at the *X–Y, X–Z*, and *Y–Z* axes did not differ between the VEH-P0 and DEX-P0 males. We further observed the PSD-95 immunofluorescence in close apposition with the GnRH neurons in the OVLT (Figure 6A). The number of the PSD-95-ir punctae on the EGFP–GnRH neurons did not differ significantly between the VEH-P0 and DEX-P0 males in the OVLT (VEH-P0: 4.7 ± 1.8, DEX-P0: 3.3 ± 1.0) and in the AHA (VEH-P0: 2.16 ± 0.65, DEX-P0: 2.65 ± 0.51) (Figure 6B).

## Discussion

This study examines the effect of maternal DEX exposure on the GnRH neuronal spine number and alterations of synaptic inputs to the GnRH neurons in P0 male offspring. Maternal DEX exposure does not alter the spine number on GnRH neurons in the MS, OVLT, and AHA region of P0 males. Maternal DEX exposure decreases the number of syn I-ir cluster in areas adjacent to the GnRH neurons within the OVLT region and the number and levels of syn I-ir punctae cluster in close contact with the GnRH neurons within the OVLT of P0 males. These results suggest that the synaptic inputs to GnRH neurons within the OVLT region are altered by DEX exposure during early developmental stage.

### Maternal DEX Exposure on Spine Density of GnRH Neurons

Filopodia protrusions are known to be involved in the formation of mature spines, synapses, and dendritic branching and growth during early developmental stage in the brain (42, 43). A small increase in the number of filopodia on the soma of GnRH neurons was observed by maternal DEX treatment in the MS, but it was not seen in the OVLT and AHA in the P0 males. Therefore, development of the filopodia protrusions in GnRH neurons may be influenced by glucocorticoid in site-specific manner during developmental stage. Different subpopulations of GnRH neurons within the hypothalamus have been identified to understand the functional positioning of these neurons in relation to reproduction, and how they are differentially regulated by various factors and neurotransmitters (10, 11). Furthermore, a subpopulation of GnRH neurons (10–20%) in rats and mice co-expresses the glucocorticoid receptor (31, 32), suggesting a possible differential effect of glucocorticoid on the GnRH neuronal function within different regions of the hypothalamus.

Studies have also shown the role of gonadal steroids in the regulation of neuronal spine density within the hippocampus and hypothalamus (44–46). Increase in the number of spiny GnRH neurons with multiple spine-like protrusions, as well as increase in the number of spines emerging from GnRH neurons during pubertal stage suggest modulation of the GnRH neuronal plasticity and function by the high levels of testosterone (12, 13). However, the number of spines on the soma and along the dendrites of GnRH neurons did not differ between the VEH-P0 and DEX-P0 males in the MS, OVLT, and AHA region. Although gestational stress and fetal DEX exposure is known to abolish testosterone surge in fetal and newborn males (26, 47, 48) and alter sexual maturation and behavior in the adult males (27, 28), spine development of early postnatal GnRH neurons may be sustained without the influence of gonadal steroid and glucocorticoid. Previous observation using GnRH immunofluorescence has shown that 10–20% of the total GnRH immunostained neurons co-expresses EGFP in P0 rats, and the total number of EGFP-expressing GnRH neurons was not altered by the maternal DEX treatment in P0 males (35). The low numbers of EGFP-expressing GnRH neurons observed in the fetal and postnatal stages of these transgenic rats suggest a lower gene transcriptional activity during early developmental stages (35). Therefore, no difference observed in the spine density of GnRH neurons between the VEH-P0 and DEX-P0 males could possibly be attributed to the lack of glucocorticoid effect on the GnRH promoter activity on the EGFP-expressing GnRH neurons during early developmental stage.

Furthermore, there was no difference observed in the number of PSD-95-ir clusters in close apposition with the EGFP–GnRH neurons of VEH- and DEX-treated P0 males in the OVLT and AHA. PSD-95 is major scaffolding protein in glutamatergic excitatory synapses (49) and dopamine-related synaptic plasticity (50). This probably suggests that maternal DEX exposure may affect the spine plasticity at the presynaptic levels of GnRH neurons.

### Maternal DEX Exposure on Synaptic Inputs to GnRH Neurons

The number of synaptic clusters in areas surrounding the GnRH neurons decreases in the OVLT of DEX-P0 males, indicating possible synaptic loss by maternal DEX exposure. Fetal glucocorticoid exposure has been shown to cause synaptic loss and function in the forebrain due to altered synaptic gene and protein expression affecting synapse formation (51–53). High density of glutamatergic synaptic terminals, vesicular glutamate transporter-2 (vGLUT2), has been observed within the OVLT/POA compared to the MS and AHA in rats (54). Indeed, high density of vGLUT2 immunoreactive puncta is colocalized with the GnRH axon terminals in the OVLT of adult male rats (55). Our previous study has demonstrated that maternal DEX exposure significantly decreases the number of EGFP–GnRH neurons in the OVLT/POA of P0 males (35), which possibly explain the reduced number of synapsin clusters within the OVLT region. This may, in part, reflect that the lower density of neurons within the OVLT that can be contacted by the neural afferents further results in the loss of synaptic clusters within the OVLT region by maternal DEX exposure.

The synaptic inputs of neural afferents to the GnRH neurons decreases within the OVLT and AHA region, but not in the rostral MS region, suggesting that only a subpopulation of GnRH neurons are affected by maternal DEX exposure depending on their location in the brain. A higher number of synaptic inputs are observed in GnRH neurons located in the OVLT/POA compared to the MS in rats (56) and primates (57), implicating regional difference in synaptic inputs to the GnRH neurons. GnRH neurons located in the OVLT/POA region are important for the preovulatory LH surge (58–60). The spread of GnRH dendritic trees into the OVLT suggest the possible regulation of the GnRH neurons by other peripheral circulating factors (61). Furthermore, the medial subpopulation of GnRH neurons predominantly expresses *N*-methyl-d-aspartate (NMDAR1) receptors, implicating direct glutamatergic signaling on GnRH neurons in the OVLT/POA region (62). Thus, a decrease in synaptic inputs to GnRH neurons in the OVLT and AHA region suggest that maternal DEX exposure affects the development of GnRH neurons during the early postnatal stage.

The average number of syn I-ir clusters detected in close proximity to GnRH neurons in P0 males (4–6 clusters per neuron) corroborates with other studies reporting the numbers of synaptic terminal using synaptic marker synaptophysin, syn I, vesicular GABA transporter (vGAT), and vGLUT2 in adult rat (13, 15, 56). Indeed, electron microscopic studies have shown that GnRH neurons receive relatively sparse synaptic inputs on the cell soma (1–3 synaptic inputs per cell) and dendritic profiles (2–3 synaptic inputs per profile) in the adult rat, in comparison with other neuronal cell types within the POA (17, 63).

However, the identity of altered synaptic neural afferents to the GnRH neurons is unknown, as the use of presynaptic marker syn I does not allow us to discriminate among the inhibitory and excitatory synapses in direct apposition with the neurons (64). The neurotransmitters that are most likely involved in the regulation of GnRH neurons are GABA and glutamate (65), since changes in their synaptic terminals and receptor subunits on GnRH neurons have been demonstrated across development and in response to gonadal steroids (15, 16, 66). Furthermore, male rat offspring, subjected to prenatal stress, have higher expression of presynaptic marker for GABA than glutamate (67), suggesting increased GABAergic inhibition in the hypothalamus. Decrease in the synaptic cluster inputs to the GnRH neurons by maternal DEX exposure could be mediated by GABA and glutamate synaptic transmission directly on the GnRH neurons, as both vGAT and vGLUT2 synaptic terminals are colocalized with syn I clusters on GnRH neurons (15). Furthermore, the absence of a difference in number of spines on GnRH neurons between DEX-P0 and VEH-P0 males suggests that spine development is not affected by maternal DEX exposure probably due to the lack of excitatory signals (67).

Other neural afferents with close apposition to the GnRH neurons modulating GnRH neuronal development and function could be affected by maternal DEX exposure. Embryonic development of catecholamine and serotonin (5-hydroxytryptamine, 5-HT) systems (68–70) modulate GnRH neuronal migration and development in rat fetus (71, 72). Close apposition of 5-HT and catecholaminergic fibers and synaptic contacts on GnRH neurons have also been demonstrated (73–75). Prenatal glucocorticoid exposure alters development of serotonergic and catecholaminergic systems (76) and decreases 5-HT and dopamine turnover in the offspring (77), suggesting that decrease in synaptic inputs to GnRH neurons could be due to modifications of neurotransmitter afferents to GnRH neurons by DEX in early stages. Additionally, early-life DEX treatment increases gonadotropin-inhibitory hormone (GnIH) mRNA and cell numbers in the hypothalamus, which could increase the inhibitory regulation of GnRH neurons through GnIH fiber contacts (30, 78). From these, it is apparent that GnRH neurons have relatively few synaptic inputs during the early stages, which continue to develop with increasing number of primary neural afferents leading to the maturation of GnRH neurons for pubertal development. Changes in afferent inputs of neurotransmitters and neuropeptides on GnRH neurons, as a consequence of maternal DEX exposure, remain to be determined across pubertal development using additional synaptic markers.

Synaptic transmission for the regulation of GnRH secretion is demonstrated through the firing of GnRH neurons by electrophysiological approaches (20, 22). Synaptic and extrasynaptic GABA_A_ receptors on GnRH neurons are regulated by neurosteroids and androgen, to suppress the downstream firing of GnRH neurons (79, 80). Thus, the decrease in synaptic inputs by prenatal DEX exposure could affect synaptic transmission to GnRH neurons in the adult stages.

In summary, maternal DEX exposure during the late gestational stages did not alter the spine development of GnRH neurons in the P0 males. However, a decrease in synaptic cluster inputs was observed within the OVLT region, and those in close contact with GnRH neurons in P0 males suggest altered neural afferent inputs to the GnRH neurons in the early developmental stage. Therefore, early-life DEX exposure could lead to the modification of GnRH neuronal activity and maturation across development, thereby reproductive dysfunction in adulthood.

## Author Contributions

Dr. WL conducted all the experiments, analyzed data, and wrote the manuscript. Ms. MI and Mr. FK conducted PSD-95 immunohistochemistry and analyzed data. Dr. TS designed the experiments, analyzed data together with Dr. WL, and edited the manuscript. Prof. IP designed the experiment with Dr. TS, discussed about all results, and edited the manuscript.

## Conflict of Interest Statement

The authors declare that the research was conducted in the absence of any commercial or financial relationships that could be construed as a potential conflict of interest.
